# Circadian Disruption Leads to Loss of Homeostasis and Disease

**DOI:** 10.1155/2011/964510

**Published:** 2012-01-24

**Authors:** Carolina Escobar, Roberto Salgado-Delgado, Eduardo Gonzalez-Guerra, Araceli Tapia Osorio, Manuel Angeles-Castellanos, Ruud M. Buijs

**Affiliations:** ^1^Departamento de Anatomía, Facultad de Medicina, Universidad Nacional Autónoma de México, 04360 Mexico City, DF, Mexico; ^2^Departamento de Biología Celular y Fisiología, Instituto de Investigaciones Biomédicas, Universidad Nacional Autónoma de México, 04360 Mexico City, DF, Mexico; ^3^Facultad de Ciencias, UASLP, 78210 San Luis Potosí SLP, Mexico

## Abstract

The relevance of a synchronized temporal order for adaptation and homeostasis is discussed in this review. We present evidence suggesting that an altered temporal order between the biological clock and external temporal signals leads to disease. Evidence mainly based on a rodent model of “night work” using forced activity during the sleep phase suggests that altered activity and feeding schedules, out of phase from the light/dark cycle, may be the main cause for the loss of circadian synchrony and disease. It is proposed that by avoiding food intake during sleep hours the circadian misalignment and adverse consequences can be prevented. This review does not attempt to present a thorough revision of the literature, but instead it aims to highlight the association between circadian disruption and disease with special emphasis on the contribution of feeding schedules in circadian synchrony.

## 1. The Relevance of Circadian Rhythms for Homeostasis

Our physiology is organized around the daily cycle of activity and sleep [1]. In the active phase, when energy expenditure is high and food and water are consumed, organs need to be prepared for the intake, processing, and uptake of nutrients.

During sleep, energy expenditure and digestive processes decrease and cellular repair takes place [1, 2]. The autonomic nervous system and hormones, especially melatonin and corticosterone, are used to transmit signals from hypothalamic and brain stem nuclei to the body in order to prepare it for the daily changes in activity, food intake, and rest. Hypothalamic structures receive information from the suprachiasmatic nucleus (SCN), the biological clock [1, 2]; this nucleus transmits 24 h time information synchronized by the light dark (LD) cycle. It is known that neurons of the SCN, even *in vitro*, maintain a 24 h rhythm of electrical activity and neurotransmitter, release [3]. Via the secretion of its neurotransmitters, the SCN transmits rhythmicity to hypothalamic structures, to the brain, and to the rest of the body. An example is the secretion of corticotrophin releasing hormone and thus the secretion of ACTH, which is modulated by the SCN via vasopressin projections to the dorsomedial hypothalamus (DMH) and subsequently to the paraventricular nucleus (PVN) [4]. Simultaneously, the SCN modifies the sensitivity of the adrenal gland for ACTH via the preautonomic sympathetic neurons of the PVN, thus creating the most efficient way to transmit its time message to the body [5]. Light influences directly the neuronal activity of the SCN and thus inhibits melatonin secretion from the pineal via preautonomic neurons of the PVN [6, 7]. Likewise, via the autonomic system, the SCN influences peripheral organs such as liver, adrenal gland, heart, and even fat tissue for daily adjustment [8, 9]; such that the physiology of the body responds optimally to support activity or sleep and to keep it coupled to the external cycles.

Light is the main “Zeitgeber” or time signal for the SCN, however, other temporal signals may also exert synchronizing effects on the biological clock and are considered “weak Zeitgebers” because they are obscured by the dominating LD cycle [10]. For the humans, social activities and social schedules function as relevant synchronizing signals that compete or can enforce the influence of the LD cycle depending on how they are scheduled [11]. As such, work and school schedules, exercise and physical activity, as well as meal time can provide additional time signals to the biological clock [11–13]. Thus, the SCN is influenced by a complex combination of temporal signals that require congruency in order to keep all functions and behavior synchronized.

## 2. Circadian Disruption

The development of modern technology has promoted a relative independency of social and work activities from the environmental LD cycle. In the last 100 years, lifestyle has changed radically due to the use of electric light that allowed to extend activities into late hours of the night [14, 15]. The proportion of individuals engaged in night work is increasing and has reached a large part of the economically active population. Also, the development of technology has provided new possibilities of amusement and recreational activities towards the sleep hours. This nocturnal life style is extremely attractive especially for young people, resulting that a large number of teenagers and young adults are awake for many hours during the night [16]. Remaining alert in the night promotes physical activity and arousal, in addition, individuals that are awake tend to eat at the moment that the biological clock indicates time to sleep [16]. During week days, school and work schedules require that individuals wake up early, which results in sleep deprivation, while weekends allow to sleep over. The constant shift of the sleep/wake schedule from weekdays to weekends is accompanied by constant shifts of general activity providing a “weak” temporal signal for the SCN. This results in misalignment of the social time from the biological clock, this condition has recently received the term of “social jet lag” [17]. The disturbed activity schedules and the consequential constant sleep deprivation lead to a disruption of the internal temporal order, to anxiety, depression, and altered behavioral performance [18, 19]. These alterations emphasize the importance of circadian synchrony for mental health. Because young people are shifting their temporal patterns in the night period, the impact of night activities on behavioral performance and mood requires more research and is becoming a topic of high priority.

Circadian misalignment is also the consequence of transmeridian traveling, which causes an abrupt change in the time schedule and a syndrome known as “jet lag.” Jet lag is the result of a slow readjustment of physiological and behavioral rhythms that shift with different speed to the new schedule [20]. The transitory loss of circadian synchrony among different tissues and with the biological clock results in loss of homeostasis and a feeling of malaise [21], general discomfort, decrement of physical and mental performance, irritability, and depression [20]. Also, gastrointestinal disorders can be seen as a by-product of food consumption at an unusual schedule [20]. This state of internal desynchrony is transitory and depends on the number of time zones that are crossed, consequently adaptation to a new external cycle can take from 4 to 10 days [22, 23].

Circadian disruption is also observed in individuals exposed to shift work or to nocturnal work schedules (night work). In such conditions, circadian fluctuations in behavioral, hormonal, and metabolic parameters are observed but their temporal relation with the external cycles is modified. The internal synchrony is affected by environmental signals that are out of phase with the daily activities of the individual; such as the exposure to light during resting hours and the forced activity and food intake when homeostatic processes indicate a need to rest [24]. This condition leads to sleepiness and disturbed performance which may lead to increase of work accidents [25]. Similar to the alterations observed in jet lag, circadian internal desynchrony is associated with gastrointestinal disorders, disturbed metabolic fluctuations, disturbed cardiovascular functions, altered menstrual cycle, and propensity to develop cancer [26–29]. Despite the fact that persons may work for many during the night, they still may be incapable to adapt to such a night work scheme [30]. Only a minority of shift workers or night workers are able to adjust spontaneously their rhythms of core body temperature, melatonin, cortisol, or prolactin [30, 31]. In shift and night workers, who do not manage to adapt, a high propensity to smoke, drink alcoholic beverages, and use stimulants has been reported [32].

An important factor promoting circadian disruption is light at night. The human biological clock is sensitive to light changes including low-intensity light exposure [33, 34]. In healthy volunteers, different light intensities, ranging from 0.03 to 9,500 lux, during the night cause in a short term an important impairment of temperature and hormonal rhythms [35]. Such evidence indicates that humans are sensitive to light intensities used for illuminating house interiors and job areas, and that such intensities are sufficient to alter the biological clock, which can cause circadian disruption and propensity to disease [14, 35].

## 3. The Consequence of Internal Desynchrony

Several aspects of modern life, as described above, provide conflicting signals out of synchrony with temporal signals transmitted by the SCN, which mainly follows the LD cycle [2]. The consequence is a disturbed phase relation of circadian fluctuations in behavioral, hormonal, and metabolic variables, leading to circadian misalignment. In the long term, circadian disruption due to shift work or chronic jet lag may result in increased mortality in male and female workers due to cardiovascular, gastric disorders, or cancer [27, 36–41]. Recently, disturbed circadian rhythms have been suggested as strong promoters of obesity and metabolic syndrome [42–44]. Therefore, it is important to develop an understanding of the impact of circadian disruption due to shifted activity schedules and light pollution on physiological systems and homeostasis.

## 4. Rodent Models of Circadian Disruption, What We Have Learnt

Animal models provide experimental and controlled conditions for further understanding the mechanisms of circadian disruption. A strategy used frequently to induce circadian disturbance is exposing rodents to constant shifts in the onset and offset of light. Shifts of 6–8 hours are scheduled once to several times per week and for as long as 3 to 6 months [45]. Shifting the LD cycle resembles the condition of transmeridian traveling and is accepted as a good model for frequent exposure to jet lag. Phase shifts, especially when the LD cycle is advanced, imply a gradual resetting of physiological and behavioral rhythms. The speed of reentrainment after a 6 h phase advance differs among the functional systems. While general activity and the skeletal muscle achieve complete adjustment after 6–10 days [46, 47] core temperature, lungs and the liver adjust faster [21, 47, 48], leading to a loss of synchrony. Depending on the frequency of the shifts and the duration of this treatment, some groups have reported disruption of behavioral and physiological rhythms [45, 48, 49], while others do not observe significant effects [50, 51]. In a long term, frequent LD shifts alter cognitive functions and neurogenesis in the hippocampus [52], they result in a weak immune response and accelerated tumor growth in the liver after exposition to a cancer promoter [37]. Even more, a recent study reported that rats exposed to frequent shifts of the LD cycle increased the ratio of body weight gain and increased abdominal fat accumulation [45]. Such detrimental effects, however, were not found when shifts were scheduled in semicircaseptan (half-weekly) cycles; in addition, in such conditions, tumor growth was diminished in male rats [51]. Further studies are needed to better understand the contribution of infradian cycles on homeostasis and disease.

Other models of circadian disruption expose rodents to short days of 20–22 h, which are incongruous with the normal endogenous 24 h period. This short photoperiod challenges the capacity of circadian system to adjust and to produce a circadian desynchrony [53]. Under such conditions, rodents exhibit two components of activity, one free-running under a long period and the second component entrained to the 22 h LD cycle. In the SCN, this protocol produces a disruption of neuronal activity, where the dorsal SCN reflects the free-running rhythm while the ventral SCN reflects the LD synchronized rhythm [53]. Rodents housed under these conditions developed dissociation of the sleep-wake cycles from the core temperature [54] as well as changes in metabolic hormones. In the brain, circadian-disrupted mice exhibit decrease of dendritic length and decreased complexity of neuronal dendritic trees in the prelimbic prefrontal cortex, associated with reduced cognitive flexibility and altered emotional responses [55].

In order to model the effects of light pollution, rodents are maintained in constant light conditions (LL). Many studies show that circadian organization can be disrupted by LL. After 2-3 weeks in LL, rodents develop arhythmicity and hamsters exhibit “splitting” of circadian locomotor activity patterns [56, 57]. In the SCN, LL uncouples individual neurons, although individually each neuron maintains its capacity to generate circadian oscillations, their cycles are out of phase from each other [58]. In rodents, exposure to constant light leads to irritability, anxiety-like and depressive-like behaviors, and deficient performance in tests for learning and memory skills [59]. At the physiological level, LL inhibits melatonin secretion, a hormone secreted during the night, which is suggested to be a signal controlled by the SCN for transmitting the night information to the rest of the body. Melatonin also signals back to the SCN possibly to fortify the night signal to the biological clock [60]. Also, constant light may affect metabolic activity, glucose utilization, and protein synthesis [61]. The absence of melatonin secretion due to LL conditions may affect and decrease the activity of the immune system, which probably leads to accelerated aging and tumor growth [62]. The effect of constant light on tumor growth is, however, not clear, in view that constant light reduced the development of mammary tumors in young female rats [63]. On the other hand, constant light leads to increased visceral adiposity, propensity to obesity, and altered cardiovascular function [64, 65] indicating that a disturbance of the function of the SCN by constant light may alter the integrity of metabolic functions. Interestingly, the disruptive effect of constant light is delayed when rats are housed in groups, suggesting that social interaction, which is a secondary “weak” synchronizer [66], partially compensates for the missing light/dark cycle.

**Figure 1:**
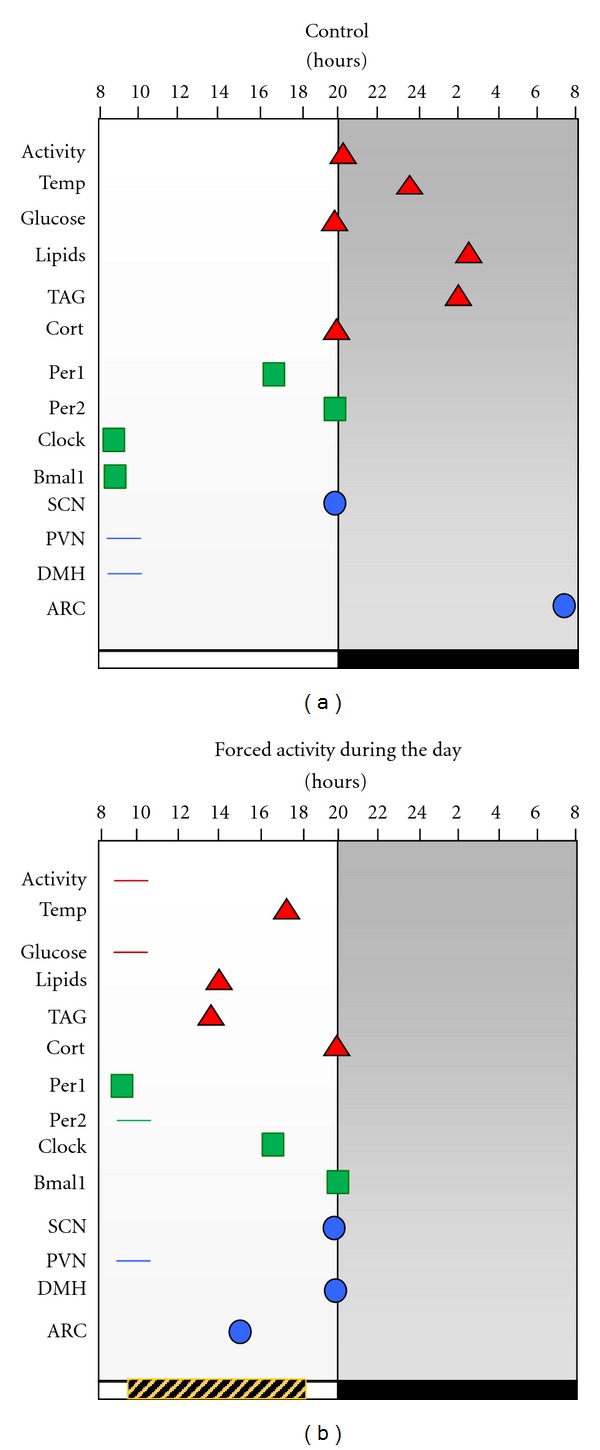
Moments of peak activity for physiological variables in rats living undisturbed in control *ad libitum* conditions (a) and rats exposed for 4 weeks to 8 h forced activity during the rest phase (b). Symbols represent the moments of maximal expression of each variable along the 24 h cycle. Data were obtained from three previous reports [60, 61, 67] and peak values were statistically different from low values of the same group according to a one way ANOVA (*P* < 0.05). Day and night are represented by white and black horizontal bars below the graphs and the time in the activity drums is represented by the grey striped horizontal bar. The “*y*” axis represents variables measured in metabolism (triangles) in the liver (squares) and in the brain (circles). Horizontal lines indicate loss of rhythmicity and therefore no significant peak value for that variable and condition. The daily scheduled activity induced shifts of several variables and led to circadian misalignment. Abbreviations: Temp: core temperature; TAG: triglycerides; Cort: corticosterone; Per1: clock gene period 1; Per2: clock gene period 2; SCN: suprachiasmatic nucleus; PVN: paraventricular nucleus in the hypothalamus; DMH: dorsomedial nucleus in the hypothalamus; ARC: arcuate nucleus.

For rodents, shifting the LD cycle does not completely mimic the conditions of the human shift workers or night workers, because night workers are awake and active during their sleep phase and thus experience conflicting signals between their biological clock and the unchanged LD cycle. In our group, we developed a rat model of “night work” based on forced activity during the light phase, which is the period when rats mainly sleep. To induce activity, rats were placed in slowly rotating drums (33 cm diameter × 33 cm long) with four concentric subdivisions, which allow individual housing. Drums rotate with a speed of one revolution/3 min and due to this speed rats can sit, groom, and even lie down, however, they cannot sleep and are forced to be awake and active. Importantly, they can eat from chow pellets and drink from a small bottle hanging from the middle tube of the drums [68]. Rats are placed in such drums daily during 8 h of the light phase, for 5 days per week (Monday to Friday) without altering their LD cycle. After 8 hours of forced activity rats are returned to their home cages, allowing them (like human shift workers) 16 h for sleep and recovery. During weekends, rats remained undisturbed in their home cages. After 4 weeks in this protocol, rats developed disturbed daily activity rhythms characterized by reduced nocturnal activity and enhanced activity during the day. Interestingly rats shifted their feeding patterns toward the light phase and a high proportion of their daily ingestion occurred during the hours spent in the drums [68]. Consequently, core temperature and metabolic rhythms were shifted to the light phase or were completely disrupted (see Figure 1). Also, body weight and abdominal fat increased in rats exposed to the drums during the light phase, in contrast, all these effects were not observed in rats exposed to activity drums in the night, which corresponds to their active phase. The neuronal activity in hypothalamic nuclei involved in feeding and activity was also shifted in this “night work” model, while the activity of the SCN remained locked to the LD cycle [69]. Consequently, this model revealed circadian desynchrony already at the level of the hypothalamus with an absence of changes in indicators of SCN activity. Also, metabolic and behavioral daily rhythms indicated a circadian misalignment.

## 5. The Contribution of Feeding Schedules for Circadian Synchrony/Desynchrony

Rodent models of circadian disruption have confirmed the relevance of circadian synchrony for homeostasis and the results of these studies suggest that loss of circadian synchrony affects physiological and metabolic congruency leading to disease and overweight. These observations are in consonance with health problems observed in individuals exposed to chronic jet-lag, to shift and night work, and to light at night, confirming that circadian misalignment is the mechanism underlying the loss of homeostasis [25, 70].

Several strategies have been tried to prevent circadian disruption and to accelerate resynchrony after a phase disturbance. Melatonin administration simultaneous to phase shifts is highly effective for the treatment of a range of symptoms that result from jet lag [21, 71]. Melatonin directly acts on the SCN and via melatonin receptors (MT1 and MT2) may reset, the biological clock, restore disturbed circadian rhythms and thus sleep disorders [72]. Arousal and enhanced locomotor activity, including scheduled exercise, have also been suggested as therapeutic strategies to accelerate circadian adjustment [67]. In addition, animal models, indicate that the beneficial effects are dependent on the time of the day when they are applied [73].

With our model of forced activity, we have demonstrated that activity during the sleep phase not only disrupts the daily activity pattern but also shifts the normal nocturnal pattern of food intake toward the working hours in the light phase. These observations indicate that when forced to be active, rodents choose to eat. This is congruent with observations in night workers, since it is well documented that shift and night works promote changes in feeding patterns, resulting in increased food intake during the working hours that coincide with the normal resting phase [74, 75]. This effect has been observed in night workers, who ingest up to 70% of their daily intake during their work hours, and it is common that they choose diets rich in carbohydrates [76]. A follow-up study with university students reported that that night-active persons develop low amplitude and desynchronous timing of endocrine metabolic rhythms, which was associated with shifted eating patterns [77]. As observed with our rodent model, several studies have confirmed that in human populations, shifting activity and the main food consumption toward the night results in propensity to obesity and increased accumulation of abdominal fat [78, 79]. In agreement with Kreier et al. [9], the increase of abdominal fat can be seen as a symptom of unbalance in metabolism and in circadian synchrony. Disturbed circadian rhythms also impact the quality and amount of sleep. Jet lag, as well as shift work, night work, and light pollution, leads to poor or short sleep, which also contributes to food ingestion at the wrong time, especially because individuals tend to ingest food while staying awake. This condition leads to metabolic alterations and overweight [80–82]. Moreover, the restriction of food consumption and activity to the active phase should prevent circadian disruption and overweight [78, 79, 83].

In rodents, circadian disruption and metabolic dysfunction can also be prevented by exposing individuals to hypocaloric and/or low-fat diets that reduce body weight. When rodents are food restricted with a hypocaloric diet, the circadian amplitude is enhanced regardless whether food is restricted to the day or night [84, 85]. Also, hypocaloric diets lower the metabolic rate and lead to healthier individuals and a longer life span [85, 86]. The mechanism underlying the influence of feeding schedules on circadian rhythms, metabolism, physiology, and life span may be associated with the potent influence of food as synchronizer of brain and peripheral oscillators [87]. In rodents, daily food restriction with a normo- or a low-caloric diet induces metabolic and digestive temporal adjustments to meal time [88, 89] as well as neural oscillations in the brain [90–93]. Glucose, ATP levels, and the redox state in the cell set cellular daily oscillations that may provide support to signals transmitted by the biological clock for peripheral entrainment [87].

In a recent study, we explored the power of food as synchronizing signal for the circadian system. With a protocol of jet lag based on a phase advance of the LD cycle, we reported that when meal time was scheduled to coincide with the new onset of activity, the days required for reentrainment were substantially reduced, especially when meal time was shifted simultaneous to the LD shift [46]. This study provided evidence that in fact feeding schedules give support to temporal signals transmitted from the SCN to the body for circadian synchrony. A similar beneficial effect was observed with our rodent model of night work, where scheduled food to the night, the normal active phase, prevented circadian disruption [88]. The relevance of keeping food intake coupled with the LD cycle as a complementary time signal was confirmed by a recent study reporting that a simultaneous shift of the feeding schedule with the LD cycle facilitated the circadian resetting of clock genes in peripheral organs [94]. On the other hand, uncoupling LD cycles from feeding schedules leads to circadian desynchrony and metabolic alterations [78, 79].

## 6. Conclusions

Aligned circadian rhythms in brain and periphery are necessary for adaptation and for homeostasis. When the circadian order is disturbed, individuals cannot produce efficient responses to daily challenges associated with the day/night cycle and in a long term develop disease. Although the main Zeitgeber adjusting the biological clock is the light/dark cycle, other temporal signals considered “weak” contribute to its daily entrainment, among them arousal and activity, exercise, temperature, and for the human, social schedules. Evidence collected in recent years demonstrate the importance of feeding schedules as a powerful entraining signal for diverse functional systems and support the significance of maintaining feeding schedules in harmony with the LD cycle and with the SCN for efficient and correct entrainment. This implies that a correct entrainment can only be achieved when all temporal signals are coupled and when feeding schedules are congruent with the light/dark cycle. The implications of these observations point out that modern life style which promotes predominantly nocturnal activities can have deleterious consequences for human physiology. This requires attention because children, teen-agers, and young adults are shifting their temporal activity pattern toward the night. Nocturnal activity, nocturnal food intake, night work, transmeridian traveling, and light during the night elicit confounding temporal signals to the biological clock and promote circadian misalignment and physiological disturbances. This nocturnal life style also induces poor sleep quality and quantity, anxiety, depression, and modified feeding patterns. When individuals are unable to correct their nocturnal habits, a possible strategy to prevent circadian misalignment is to couple feeding schedules to the day. According to the evidence discussed in this review, this strategy will avoid internal circadian disruption and may be useful to prevent disease. The mechanisms underlying this beneficial effect on physiology, behavioral performance, and mood require more research and should become a topic of high priority in the area of circadian physiology.
